# Five distinct biological processes and 14 differentially expressed genes characterize *TEL/AML1*-positive leukemia

**DOI:** 10.1186/1471-2164-8-385

**Published:** 2007-10-23

**Authors:** Virginie Gandemer, Anne-Gaëlle Rio, Marie de Tayrac, Vonnick Sibut, Stéphanie Mottier, Béatrice Ly Sunnaram, Catherine Henry, Annabelle Monnier, Christian Berthou, Edouard Le Gall, André Le Treut, Claudine Schmitt, Jean-Yves Le Gall, Jean Mosser, Marie-Dominique Galibert

**Affiliations:** 1 CNRS UMR 6061 Laboratoire de Génétique et Développement, Equipe Régulation transcriptionnelle et oncogenèse, Université de Rennes-1, Faculté de Médecine, IFR140 GFAS, 2 av du P^r ^Léon Bernard, CS 34317, 35043 Rennes cedex, France; 2 Department of Biochemistry and Molecular Genetics, Medical Genomic Unit, CHU Rennes, France; 3 Department of OncoPediatrics, CHU Rennes, France; 4 Laboratory of Hematology Rennes, France; 5 Laboratory of Cytogenetics, CHU Rennes, France; 6 Ouest-Genopole ^®^, transcriptomic platform, IFR 140, Rennes, France; 7 Laboratory of Hematology, CHU Brest, France; 8 Department of OncoPediatrics, CHU Nancy, France

## Abstract

**Background:**

The t(12;21)(p13;q22) translocation is found in 20 to 25% of cases of childhood B-lineage acute lymphoblastic leukemia (B-ALL). This rearrangement results in the fusion of *ETV6 *(*TEL*) and *RUNX1 *(*AML1*) genes and defines a relatively uniform category, although only some patients suffer very late relapse. *TEL/AML1*-positive patients are thus an interesting subgroup to study, and such studies should elucidate the biological processes underlying TEL/AML1 pathogenesis. We report an analysis of gene expression in 60 children with B-lineage ALL using Agilent whole genome oligo-chips (44K-G4112A) and/or real time RT-PCR.

**Results:**

We compared the leukemia cell gene expression profiles of 16 *TEL/AML1*-positive ALL patients to those of 44 *TEL/AML1*-negative patients, whose blast cells did not contain any additional recurrent translocation. Microarray analyses of 26 samples allowed the identification of genes differentially expressed between the TEL/AML1-positive and negative ALL groups. Gene enrichment analysis defined five enriched GO categories: cell differentiation, cell proliferation, apoptosis, cell motility and response to wounding, associated with 14 genes -*RUNX1, TCFL5, TNFRSF7, CBFA2T3*, *CD9*, *SCARB1, TP53INP1, ACVR1C, PIK3C3, EGFL7*, *SEMA6A, CTGF, LSP1, TFPI *– highlighting the biology of the *TEL/AML1 *sub-group. These results were first confirmed by the analysis of an additional microarray data-set (7 patient samples) and second by real-time RT-PCR quantification and clustering using an independent set (27 patient samples). Over-expression of *RUNX1 (AML1) *was further investigated and in one third of the patients correlated with cytogenetic findings.

**Conclusion:**

Gene expression analyses of leukemia cells from 60 children with *TEL/AML1*-positive and -negative B-lineage ALL led to the identification of five biological processes, associated with 14 validated genes characterizing and highlighting the biology of the *TEL/AML1*-positive ALL sub-group.

## Background

Acute lymphoblastic leukemia (ALL) is the most common childhood malignancy and is diagnosed in about 500 children in France every year [1]. Most of these malignancies (80%) involve the B lineage (B-ALL) and children have a good expected outcome on most treatment protocols. However, B-ALL is a heterogeneous disease, and therapeutically relevant standard and high-risk group categories (SR and HR) have been defined according to characteristics at diagnosis (age, white blood-cell counts, central nervous system involvement, and cytogenetic abnormalities of the leukemia cell clone). In pediatric ALL, the t(12;21)(p13;q22) chromosomal translocation is the most frequent and is found in about 25% of B-ALL cases; this translocation involves the fusion of the *ETV6 *(*TEL*) and *RUNX1 *(*AML1*) genes. Most treatment protocols result in a good outcome for *TEL/AML1*-positive ALL patients, but *TEL/AML1 *translocation is not currently used as a stratifying marker in most therapeutical protocols [2-5]. Thus, clinical features of *TEL/AML1*-positive patients determine whether they are in the SR or HR category. The *TEL/AML1 *fusion may therefore not be the single key molecular event causing leukemia spread, and if this is the case, *TEL/AML1*-positive ALL might involve other critical gene modifications; any such modifications remain to be documented [6,7].

DNA-based microarrays can be used to study the expression levels of thousands of genes and to screen genes with different expression profiles in a single experiment. We therefore used this method to investigate the molecular pathways characterizing *TEL/AML1*-positive leukemia. This approach, using either one-color (Affymetrix) or two-color (NCBI) microarray technologies, has been widely used for refining the diagnosis of ALL and for predicting the response of ALL patients to treatment [8-10]. The first studies described gene expression patterns that could be used to distinguish leukemic blast lineages [11,12], and subsequent studies identified various gene expression signatures characterizing relevant clinical leukemia subtypes, in particular the *E2A/PBX1, BCR/ABL, TEL/AML1 *and *MLL *rearrangements [13-15].

We carried out a prospective multicentric study on childhood B-ALL leukemia to elucidate the molecular processes involved in *TEL/AML1*-positive leukemia. All the patients included in this study received treatment according to the French FRALLE 2000 trial. We used Agilent whole-genome oligo-chips (44K-G4112A) to compare the gene expression signatures of *TEL/AML1-*positive patients to those of *TEL/AML1*-negative patients with no recurrent chimeric products irrespective of their clinical risk category. Previous microarray gene expression studies [13-15] had revealed the effect of chromosomal alteration on transcription profiles, so we excluded from our cohort those patients with other recurrent chromosomal translocations or fusion transcripts (*BCR/ABL, E2A/PBX1, MLL *rearrangements). We then searched for the biological pathways associated with genes differentially expressed in *TEL/AML1-*positive leukemia (*ETV6/RUNX1*).

## Results

### Patient selection

Sixty patients with B-lineage ALL who were treated between 2002 and 2005 according to the FRALLE 2000 protocol were included in the study. The clinical and biological characteristics of the 60 patients are summarized in Table 1. Microarray data were obtained for 33 patients; the patients were grouped into two sets (Set-A and B) according to inclusion date. Genes of interest were selected using data Set-A and tested using data Set-B. Set-A comprised 26 patients included over the three-year period up to December 2004: 19 of these patients, including six presenting a *TEL/AML1 *rearrangement, were in the standard-risk group (SR), and seven, including one presenting a *TEL/AML1 *rearrangement, belonged to the high-risk group (HR). Set-B comprised seven patients included in 2005: five, including one presenting a *TEL/AML1 *rearrangement, belonged to the SR group; and two, including one presenting a *TEL/AML1 *rearrangement, belonged to the HR group. In addition to microarray investigations with these patients, we performed Quantitative-RT-PCR with samples from an independent set of 27 patients (Set-C), including seven *TEL/AML1*-positive patients. None had CNS involvement. With the exception of two *TEL/AML1*-negative patients (SR-52 and HR-58), all patients were good early responders to treatment, and other than the *TEL/AML1*-negative HR patient 21, all Set-A patients were in a first complete remission phase, with a median follow-up of 46 months, at the time of the study.

**Table 1:** Characteristics of the patients of the three sets (n = 60)

Patient	Sex	Age at diagnosis (years)	WBC (Count × 10^9^/l)	Recurrent Rearrangement	Risk group
***Set A***					

1	F	5	83.1	none	HR
2	F	7	21.6	none	SR
3	M	2.5	4.5	*TEL/AML1*	SR
4	F	1.5	1.2	none	SR
5	M	4	18.5	*TEL/AML1*	SR
6	F	2.5	52.5	none	HR
7	F	2	5.4	none	SR
8	F	8	0.9	none	SR
9	M	3	7.8	none	SR
10	M	3	5.4	none	SR
11	F	2	7.2	none	SR
12	F	6	18.4	*TEL/AML1*	SR
13	F	4	24.3	*TEL/AML1*	SR
14	F	5	4.5	none	SR
15	M	13	3.9	none	HR
16	F	2	3.6	none	SR
17	M	4	47	*TEL/AML1*	SR
18	M	10	35.8	*TEL/AML1*	HR
19	F	3	14.9	none	SR
20	F	14	82	none	HR
21	M	6	68	none	HR
22	F	10	150	none	HR
23	M	2	15.4	*TEL/AML1*	SR
24	M	3	2.9	none	SR
25	M	3	5.4	none	SR
26	F	3	41	none	SR

***Set B***					

27	F	7.5	4	none	SR
28	M	6	26.2	none	SR
29	M	15	4.2	none	HR
30	F	2	2.2	none	SR
31	F	3	22.6	none	SR
32	M	2	130	*TEL/AML1*	HR
33	M	2.5	1.3	*TEL/AML1*	SR

***Set C***					

34	F	9	1.4	none	SR
35	M	5	12.1	*TEL/AML1*	SR
36	M	1	34.3	none	SR
37	F	6	17.9	none	SR
38	M	3	3.3	none	SR
39	F	3	7.3	none	SR
40	F	6	1.9	*TEL/AML1*	SR
41	F	6	37.5	*TEL/AML1*	SR
42	M	5	4.2	none	SR
43	F	6	38.7	*TEL/AML1*	SR
44	F	6	2.7	none	SR
45	F	3	13	none	SR
46	M	5	1.3	none	SR
47	M	3	16.5	*TEL/AML1*	SR
48	M	3	6.8	*TEL/AML1*	SR
49	F	5	9.8	none	SR
50	M	1	6.8	none	SR
51	M	7	36.9	none	SR
52	M	2	1.6	none	SR
53	M	7	13.5	*TEL/AML1*	SR
54	M	9	24	none	SR
55	F	3	10.2	none	SR
56	F	6	9.3	none	SR
57	M	3	6.9	none	SR
58	M	10	96	none	HR
59	M	6	1.4	none	SR
60	M	3.5	83.6	none	HR

WBC: white blood cell count; F:Female, M:Male

HR and SR are respectively high risk and standard risk groups according to the FRALLE 2000 trial.

• HR: age ≥ 10 years, or white blood cell count ≥ 50 × 10^9^/l, or central nervous system involvement, or t(9;22), or t(4;11) or MLL rearrangement, or hypoploidy (≤ 44 chromosomes)

• SR: 1 ≤ age < 10 years, and white blood cell count < 50 × 10^9^/l, and no central nervous system involvement, and none of the following cytogenetic features: t(9;22), t(4,11) or MLL rearrangement, hypoploïdy

• *TEL/AML1*-positive ALL and *TEL/AML1*-negative ALL can be assigned to HR or SR categories, according to clinical features.

### Gene expression patterns clearly distinguished *TEL/AML1*-positive leukemia from leukemia without recurrent chromosomal abnormalities

**Figure 1:**
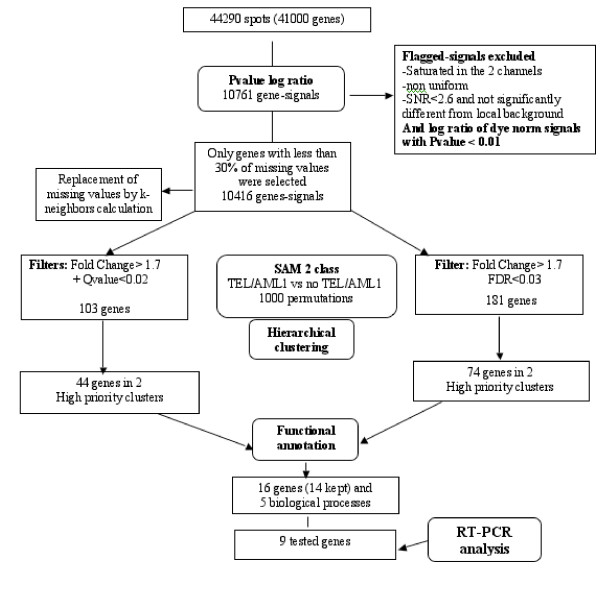
**Analysis flowchart: overview of the strategy used for gene selection using Set-A microarray data**. At each level, the data set was filtered to remove genes that showed poor robustness and no significant difference in expression level between the *TEL/AML1*-positive and the *TEL/AML1*-negative ALL subclasses. SAM denotes Significant Analysis of Microarrays. Two groups of genes (according to the filters applied) were selected and functionally annotated. Nine of the 16 genes were selected for RT-PCR validation on the basis of their biological relevance.

**Figure 2:**
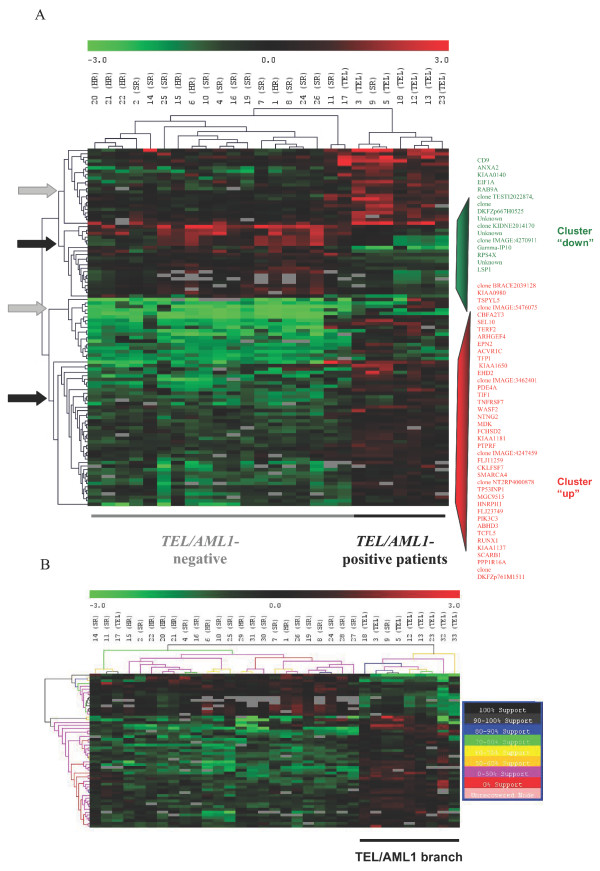
***TEL/AML1 *gene expression signature of Set-A patients**. (A) Two-class SAM was applied to Set-A data. The data were filtered on the basis of a difference greater than 1.7 fold and a Q value less than 2%, leaving 103 clones (1000 permutations, median false positive = 4) associated with a *TEL/AML1 *signature. Clustering analysis of Set-A patients segregates *TEL/AML1*-positive patients together, except for patient 17 who clusters with patient 11 in a distinct branch and for patient 9 who does not present a *TEL/AML1 *fusion transcript, and segregates with *TEL/AML1*-positive ALL. Gene expression is visualized, with green and red representing down and up-regulated genes, respectively. Gray corresponds to missing data (absence of signal) as described in "Patients, Materials and Methods". The color scale above the dendrogram extends from 0.125 to 8.0 times the mean (-3 to +3 in log2 space). Two gene clusters (indicated by black arrows), consisting of either up-regulated genes or down-regulated genes, differentiate *TEL/AML1*-positive and -negative ALL. Gray arrows indicate the branches, which were unable on their own to segregate *TEL/AML1 *positive patients. (B) Support tree of Set-A and Set-B patients using the 55 clones (44 distinct genes) identified by the clustering analysis. Resampling with replacement was conducted on experiments and genes for 100 iterations. The branches of the resulted tree are colorized to denote the percentage of times a given node was supported over the resampling trials. Two branches still distinguished *TEL/AML1*-positive ALL from *TEL/AML1*-negative ALL with 100% reproducibility when Set-B samples have been added to Set-A. Two stable clusters of genes (up- and down-regulated genes) were identified and further explored by functional analysis.

To identify genes with expression profiles that differentiate *TEL/AML1*-positive patients from *TEL/AML1*-negative patients with no recurrent chromosomal abnormalities we used data Set-A. We selected 10761 gene signals that displayed log-ratio intensities that were significantly different from the mean (PvalueLogRatio < 0.01) for at least one array. Only 10416 gene signals, those present on at least 70% of the Set-A arrays, were retained for SAM analysis. Two preliminary two-class SAM were performed, comparing *TEL/AML1*-positive patient data to either *TEL/AML1*-negative HR or SR patient data to assess specific sample behavior in a more homogeneous situation than the pooled *TEL/AML1*-negative HR and SR group [16]. Interestingly, we found that patient 18, the only *TEL/AML1*-positive HR patient (who was initially excluded from SAM analysis because he belonged to both groups) segregated with the *TEL/AML1*-positive SR group and not with the HR patient group. This is consistent with the fusion transcript affecting the gene expression profile (data not shown). Similarly, we found that patient 9, a *TEL/AML1*-negative patient, clustered within the *TEL/AML1 *branch and that patient 17, a *TEL/AML1*-postive patient, did not segregate into the *TEL/AML1 *branch. RT-PCR, using a different set of primers in a different laboratory, confirmed the presence of a *TEL/AML1 *transcript in patient 17 and its absence from patient 9; this indicates some heterogeneity in the TEL/AML1 group (data not shown). To avoid heterogeneity bias associated with particular patients and to highlight general processes, patients 9 and 17 were withdrawn from the two-class SAM gene-selection step comparing the *TEL/AML1*-positive group with the *TEL/AML1*-negative group. However, the data for these two patients were included in the final HC representation. To increase the robustness of gene-selection step, two cut-offs were applied successively, consistent with a 1.7-fold change, to exclude genes that varied little between the samples, (FC > 1.7), with or without a Q-value filter (Qvalue < 0.02) limiting the number of genes selected (Figure 1). This gave two *TEL/AML1 *gene sets, one of 181 genes and the other of 103 genes. These gene sets were used for hierarchical clustering representation of patient Set-A. Because the Set-A cohort was small, we validated our second selection (181 genes) by estimating the Benjamini and Hochberg false discovery rate (FDR): this value was consistently low (3.19% indicating only six false positives among the 181 genes designated as significant, data not shown). Both gene sets (181; 103) were able to cluster the *TEL/AML1*-positive Set-A patients, other than patients 9 and 17, in one branch. Hierarchical clustering highlighted 74 distinct genes among the 181 selected and they were separated into two clusters (data not shown). With the group of 103 selected genes, hierarchical clustering highlighted two clusters (44 distinct genes) able to group *TEL/AML1-*positive ALL (each cluster corresponds to a single branch of the hierarchical tree in which intercluster distance directly correlates with dissimilarity in gene expression) (Figure 2A). A resampling-based procedure (bootsrapping) was used to assess reproducibility (data not shown). Only these two restricted gene sets, of 74 and 44 genes, which correspond to a different profile of expression behavior associated with the presence of a *TEL/AML1 *chromosomal rearrangement, were able on their own to cluster the *TEL/AML1 *Set-A patients as previously and the *TEL/AML1 *Set-B patients into one branch. They were also able to segregate, with 100% reproducibility, the *TEL/AML1 *Set-A and -B patients together in the same branch, with the exceptions of patients 9 and 17 (Figure 2B).

### Identification of genes characterizing pathways specific to *TEL/AML1*-positive lymphoblasts

**Figure 3:**
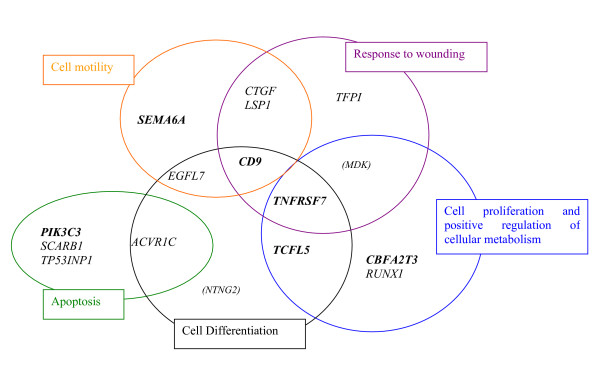
**Schematic representation of selected genes for *TEL/AML1 *with associated enriched GO terms**. Representation of enriched GO term analysis (p < 0.05) obtained by comparison of the *TEL/AML1 *gene set to the Webgestalt pre-stored human genome gene set. Each circular area represents groups of genes, sharing common properties within relevant biological processes. Five discrete enriched GO categories are identified: cell differentiation, cell proliferation, apoptosis, cell motility and response to wounding. Enriched GO categories are represented by 16 annotated genes. Six (in bold) of these 16 genes had been previously identified, and two, in brackets, were not used for further analysis because of their tissue-specific expression patterns.

We investigated the molecular pathways depicting *TEL/AML1*-positive lymphoblasts by characterizing the *TEL/AML1 *gene sets of 74 and 44 genes identified above according to their gene ontology (GO) annotations. Five enriched GO categories were revealed: cell differentiation, cell proliferation, apoptosis, cell motility and response to wounding. Cell motility only concerned the set of 74 genes, whereas the other biological processes were obtained with both 74 and 44 gene sets. Sixteen annotated genes were associated with these biological processes (Figure 3): the literature indicates that 14 genes may be involved in B lymphoblast cell biology, but that the other two genes, *MDK *and *NTNG2*, are not. Both these genes are mainly expressed in the nervous system. Their expression in *TEL/AML1*-positive B-lymphoblast cells may thus be a consequence of the alteration of cell differentiation and proliferation process rather than indicating that they have a particular function in the TEL/AML1 process. Therefore, we did not include them in subsequent analyses and focused on the 14 apparently biologically relevant genes (Table 2). Over-expression of *RUNX1, CBFA2T3, TCFL5, TNFRSF7 *and concomitant under-expression of *CD9 *characterize cell proliferation and differentiation processes in the hematopoietic lineages. Over-expression of the *SCARB1, TP53INP1, ACVR1C *and *PIK3C3 *genes correlates with cell survival. Over-expression of the *EGF7*, *SEMA6A *and *CTGF *genes with concomitant under-expression of the *LSP1 *and *CD9 *genes is characteristic of cell migration and response to wounding.

**Table 2:** Genes selected for biological analysis

**Gene name [Representative public ID]**	**Fold Change**	***q *value**
***Genes overexpressed in TEL/AML1 positive ALL***		

Scavenger receptor class B, member 1 (**SCARB1**) [NM_005505]	2.12	< 0.01
Tumor necrosis factor receptor superfamily, member 7 **(TNFRSF7**) [NM_001242]	1.99	< 0.01
Tissue factor pathway inhibitor (lipoprotein-associated coagulation inhibitor) **(TFPI**) [NM_006287]	2.71	< 0.01
Activin A receptor, type IC (**ACVR1C**) [NM_145259]	1.95	< 0.01
Tumor protein p53 inducible nuclear protein 1 (**TP53INP1**) [NM_033285]	2.53	< 0.01
Core-binding factor, runt domain, alpha subunit 2; translocated to, 3 (**CBFA2T3**) [NM_005187]	3.45	< 0.01
Runt-related transcription factor 1 (acute myeloid leukemia 1; aml1 oncogene) **(RUNX1**) [NM_001754]	1.75	< 0.01
cDNA FLJ14565 fis, clone NT2RM4000233, highly similar to Mus musculus **semaphorin VIa**. [AK027471]	2.60	< 0.01
Transcription factor-like 5 (basic helix-loop-helix) (**TCFL5**) [NM_006602]	2.85	< 0.01
Phosphoinositide-3-kinase, class 3 (**PIK3C3**) [NM_002647]	2.17	< 0.01
Connective tissue growth factor (**CTGF**) [NM_001901]	1.93	0.023
EGF-like-domain, multiple 7 **(EGFL7**) [NM_016215]	1.81	0.039

***Genes underexpressed in TEL/AML1 positive ALL***		

CD9 antigen (p24) (**CD9**) [NM_001769]	3.72	< 0.01
Lymphocyte-specific protein 1 (**LSP1**) [NM_002339]	2.82	0.017

The expression of all the selected genes differs by than 1.7-fold between *TEL/AML1*-positive ALL and *TEL/AML1*-negative ALL groups.

### Validation of biologically relevant genes

**Figure 4:**
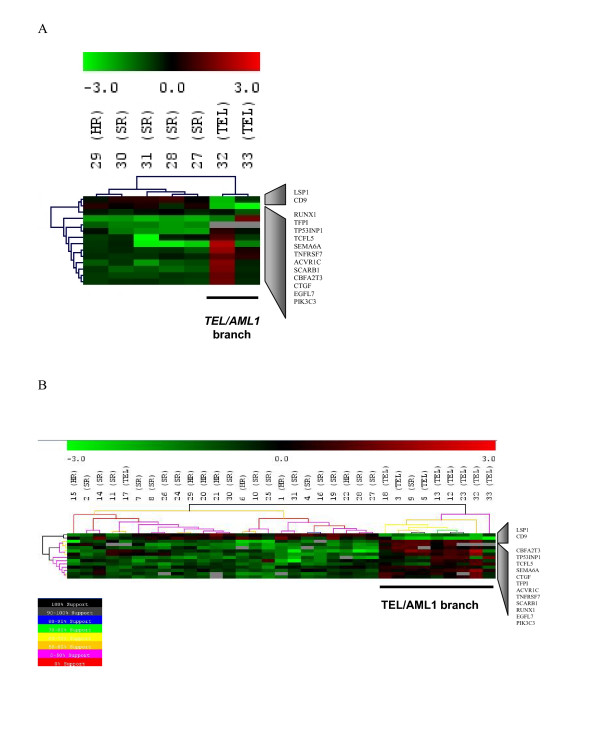
**Validation of the selected genes for *TEL/AML1 *using Set-B microarray data**. (A) Hierarchical clustering analyses (Euclidean distance and average linkage) of Set-B microarray data using the 14 selected genes for *TEL/AML1*. Patients are segregated according to the presence or absence of the *TEL/AML1 *rearrangement. (B) Support tree of Set-A and Set-B patients using the 14 selected genes for *TEL/AML1*. Two branches clearly distinguished *TEL/AML1*-positive ALL and *TEL/AML1*-negative ALL with 100% of reproducibility when resampling with replacement was conducted on experiments and genes for 100 iterations. The expression levels of the RUNX1 gene can explain the clustering of patients 9 and 17.

We validated the microarray results in two steps. First, we used a new microarray data set (Set-B) to perform clustering analysis based on the 14 selected genes (*RUNX1, TCFL5, TNFRSF7, CBFA2T3*, *CD9*, *SCARB1, TP53INP1, ACVR1C, PIK3C3, EGFL7*, *SEMA6A, CTGF, LSP1, TFPI*). Confirming the Set-A results, the *TEL/AML1*-positive ALL patients were grouped together in one branch (Figure 4A) separate from *TEL/AML1*-negative ALL patients, whose blast cells did not contain any recurrent chromosomal translocation. Additionally, hierarchical clustering and bootstrapping of data for Set-A and Set-B patients, using these 14 genes, satisfactorily segregated the *TEL/AML1*-positive patients into one branch (Figure 4B). As in the analysis described above, patient 9 (*TEL/AML1*-negative) and patient 17 (*TEL/AML1*-positive) did not classify according to their chromosomal rearrangement.

**Figure 5:**
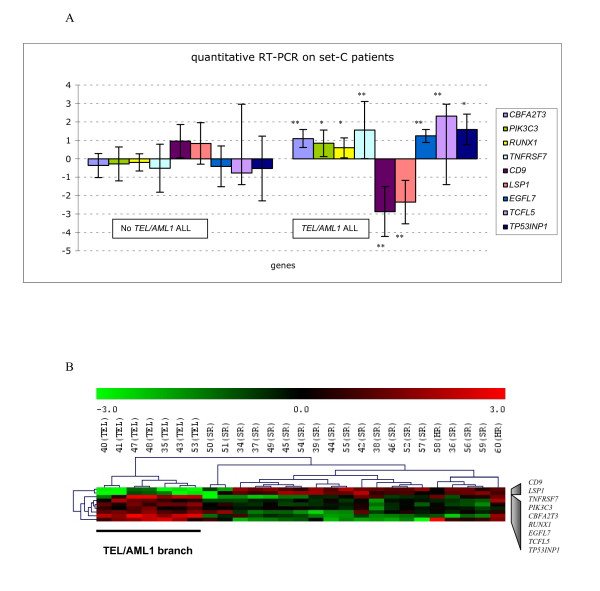
**Validation of the selected genes for *TEL/AML1 *by quantitative RT-PCR using the independent Set-C patients**. (A) Expression in log2, of mean relative levels of *TCFL5, PIK3C3, CBFA2T3*, *TNFRSF7, RUNX1, EGFL7, TP53INP1, LSP1 *and *CD9 *in *TEL/AML1*-positive (n = 7) and *TEL/AML1*-negative (n = 20) Set-C samples.*LSP1 *and *CD9 *are significantly (*P *< 0.01) under-expressed in *TEL/AML1*-positive ALL patients and each of the seven other genes is significantly (with either *P *< 0.01* or *P *< 0.05**) over-expressed in *TEL/AML1*-positive ALL patients; these findings agree with microarray data obtained with Set-A and Set-B patients. (B) Hierarchical clustering analysis (Euclidean distance and complete linkage) of Set-C patients using quantitative RT-PCR data for the nine tested genes. The dendrogram clearly distinguishes *TEL/AML1*-positive patients from *TEL/AML1*-negative patients.

Second, gene expressions were quantified using real-time RT-PCR with the independent Set-C patients. Nine genes (*TCFL5, PIK3C3, CBFA2T3*, *TNFRSF7, RUNX1, EGFL7, TP53INP1, LSP1 *and *CD9*) were chosen from the 14 selected above as being the most relevant biologically and able on their own to segregate Set-A and -B patients into appropriate clusters (*TEL/AML1*-positive versus *TEL/AML1*-negative; data not shown). Despite the genetic variability observed within each group (highlighted by the SD values), the mean gene expression values were significant according to Student's t-test, with either a *P *< 0.05 or < 0.01. *TCFL5, PIK3C3, CBFA2T3, RUNX1, EGFL7, TP53INP1 *and *TNFRSF7 *were over-expressed and *CD9 *and *LSP1 *under-expressed in the *TEL/AML1*-positive ALL relative to the *TEL/AML1*-negative subgroup, consistent with the microarray findings (Figure 5A). Hierarchical clustering of Set-C patient data using these nine genes segregated *TEL/AML1*-positive patients into one distinct branch (Figure 5B).

### Relationship between microarray data and cytogenetic data

We then examined the cytogenetic data to document *TEL/AML1 *(*ETV6/RUNX1*) patient clustering further. FISH analysis revealed that patient 9, who did not display a t(12;21) translocation but systematically clustered with the *TEL/AML1 *branch, presented a tetrasomy of the *AML1 (RUNX1) *gene (Table 3). This over-represented *RUNX1 *gene is consistent with the over-expression of *RUNX1 *seen on the microarray (log_2_-ratio = 0.59). Similarly, patient 17, who displayed a t(12;21) translocation and systematically clustered with the *TEL/AML1*-negative subgroup, had a low level of *RUNX1 *expression (log_2_-ratio = -1.31) and only two *RUNX1 *gene copies (FISH), even though three chromosomes 21 were detected (karyotype and chromosome painting) (Table 3). FISH analysis of the remaining *TEL/AML1*-positive patients (3, 5, 12, 13, 18, 23, 35, 40, 41, 43, 47, 48 and 53) revealed the presence of three *RUNX1 *gene copies in three patients (3, 13 and 23). Patients 3, 5, 13, 17, 18, 40 and 43 presented a deletion of the native *TEL*.

**Table 3:** Cytogenetic and molecular data for the 16 *TEL/AML1 *(*ETV6/RUNX1*)-positive patients

Patients	*TEL/AML1 *(*ETV6/RUNX1*) Fusion gene	RISK GROUP	KARYOTYPE	AML1(*RUNX1*) Copy-Number FISH	TEL(*ETV6*) Deletion FISH
3	*TEL/AML1*	SR	45, XY, der(3)t(3;8),-8, add(12)p?12?13	3	YES
5	*TEL/AML1*	SR	46, XY	2	YES
9	NONE	SR	53, XXY,+6,+10,+10,+14,+18,+?i(21q)[16]/46, XY[7]	4	NO
12	*TEL/AML1*	SR	46, XY	2	NO
13	*TEL/AML1*	SR	49, XX,+10,+15,+der(21)[1]/49, XX, idem, del(12)[6]/50XX, idem,+18[15]/50XX, idem, del(12),+18[2]/46XX[6]	3	YES
17	*TEL/AML1*	SR	47, XY, add(8p),-10, del(11q),+21,+mar[18]/46XY[4]	2	YES
18	*TEL/AML1*	HR	46, XY, add(19)(p or q)	2	YES
23	*TEL/AML1*	SR	50, XY,+8,?der(12),+21,+mar[4]/46XY)[26]	3	NO
32	*TEL/AML1*	HR	47, XY, t(3;14)(q?13;q?23),+21[18]/46, XY)[1]	4	NO
33	*TEL/AML1*	SR	46, XY	2	NO
35	*TEL/AML1*	SR	46, XY	2	NO
40	*TEL/AML1*	SR	45, X,-X, del(6)(q12qter)	2	YES
41	*TEL/AML1*	SR	failure	2	NO
43	*TEL/AML1*	SR	46, X,?del(Xq)[20]/46, XX[16]	2	YES
47	*TEL/AML1*	SR	46, XY	2	NO
48	*TEL/AML1*	SR	46, XY[25]	2	NO
53	*TEL/AML1*	SR	46, XY, t(1;8)?(q31;q24)t(X;17)?(q21;p13) [20]/46, XY[3]	2	NO

FISH experiments determined *AML1 *(*RUNX1*) gene copy number and revealed whether or not native *TEL *(*ETV6*) was deleted. Increased *AML1 *copy number was scored when present in more than 50% of the cell population.

## Discussion

The pediatric *TEL/AML1*-positive B-ALL subgroup displays fairly uniform clinical features, making it appropriate for studying the development of this sub-type of ALL. Comparison of gene expression profiles in *TEL/AML1*-positive patients with those in *TEL/AML1*-negative patients, whose blast cells do not contain any recurrent chromosomal rearrangement, is potentially informative about the molecular processes and pathogenesis of *TEL/AML1*. We first obtained microarray data for 26 B-ALL patients included in our prospective study. These patients constituted a homogeneous group, receiving an identical treatment according to the FRALLE 2000 trial. Gene expression analysis followed by gene enrichment analysis allowed us to identify five discrete enriched GO categories – cell differentiation, cell proliferation, apoptosis, cell motility and response to wounding – that highlighted the *TEL/AML1 *biological processes. The GO categories identified were associated with 14 annotated genes (*RUNX1, TCFL5, TNFRSF7, CBFA2T3*, *CD9*, *SCARB1, TP53INP1, ACVR1C, PIK3C3, EGFL7*, *SEMA6A, CTGF, LSP1, TFPI*); the expression patterns of these selected genes allowed clustering of the *TEL/AML1*-positive Set-A patients into one branch. The expression patterns of these genes, as assessed either by microarray experiments or real-time RT-PCR, were also able to cluster the *TEL/AML1*-positive patients of two independent sets, Set-B and -C, into one branch. Thus, even though the size of the initial set was relatively small, the filters applied were stringent enough to limit the number of false positives, leading to accuracy and subsequent validation of the 14 annotated genes. Furthermore, six of the 14 *TEL/AML1*-selected genes (*TNFRSF7, CD9, TCFL5, PIK3C3, CBFA2T3, SEMA6A*) had previously been reported to be associated with *TEL/AML1 *signatures found in more heterogeneous groups of ALL patients (including those with T-ALL, Bcr-Abl, E2A-PBX, or MLL) [13,15]. The identification of the same genes through different experimental approaches (Agilent, Affymetrix, NCBI) and in different patient sets is a strong argument for their importance in *TEL/AML1-*positive leukemia process [17], and for the relevance of the additional eight newly identified genes (*RUNX1, SCARB1, TP53INP1, ACVR1, EGFL7*, *CTGF, LSP1, TFPI*). Some genes previously described as relevant, including *TERF2 *and *EPOR*, did not appear among the genes we selected. This might be due to differences in patient sets (we did not include hyperdiploid patients, with > 50 chromosomes), or to differences in the affinities of the probe sets used [14,18]. Our findings reveal new target genes characterizing limited and specific biological pathways associated with *TEL/AML1 *pathogenesis. Further *in vivo *and *in vitro *investigations to assess their biological effects should contribute to a better understanding of the disease.

Models of ALL pathogenesis have suggested that two classes of cooperating mutations are required for acute leukemia to develop [19]: one involved in impairment of differentiation and the other in cell proliferation and/or survival. We found that differentiation was not inhibited in *TEL/AML1*-positive ALL patients but, rather, was enhanced and characterized by the over-expression of differentiation genes (*TCFL5, TNFRSF7, ACVRIC*). This is in agreement with the report by Pine *et al *[6] that *TEL/AML1 *fusion preceded differentiation to pre-B cells and suggests that *TEL/AML1 *fusion occurs in a totipotent hematopoietic progenitor cell and directs cell differentiation towards the B-lineage. We also highlighted the activation of proliferation/survival oncogenic processes with the up-regulation of the *RUNX1, CBFA2T3, PIK3CT, SCARB1 *and *TP53INP1 *genes. Our study also implicated cell motility and response to wounding processes in the *TEL/AML1 *cluster. Cell migration capacity may be a clue to explaining the very late relapse events, which affect some *TEL/AML1*-positive ALL patients. Indeed, the good outcome expected for *TEL/AML1*-positive ALL children is offset by the relatively high rate of very late relapse, especially in non-hematopoietic sites such as the ovary [20,21].

Additional genetic changes are very common in *TEL/AML1*-positive ALL patients; about 70% also present with deletion of the second *TEL *gene (*ETV6*) on the non-rearranged chromosome 12 [21,22]. About half *TEL/AML1*-positive patients (7/16) displayed an additional loss of the *TEL *gene, suggesting that there may be other genetic abnormalities acting as secondary events for *TEL/AML1 *leukemogenesis or contributing to the outcome. Unlike the *TEL *gene, the *AML1 *gene (also named *RUNX1 *according to the HUGO nomenclature) was significantly over-expressed in the *TEL/AML1 *cluster. RUNX1 is a member of the Runt transcription factor family and targets key regulators of the hematopoiesis process (M-CSF R, IL3, neutrophil elastase, MPO, granzyme B, TCRs, and B-Cell receptors) through its DNA-binding domain [23]. The transcriptional activity of RUNX1 depends on its dimerization with the non-DNA binding factor CBFβ, and on the recruitment of co-factors. The RUNX1 transcription complex thus acts either as a transcriptional activator or as a repressor depending on the nature of the co-factors. The TEL/AML1 fusion protein (ETV6/RUNX1) associated with the t(12;21) translocation acts as a repressor. However, few of the genes selected in our analysis were down-regulated. This suggests that either gene up-regulation is an indirect process, dependent on the down-regulation of transcriptional repressors mediated by the TEL/AML1 fusion protein, or that the repressor function of the TEL/AML1 fusion protein is counterbalanced by the presence of a normal RUNX1 protein. This later possibility is supported by our observation of *RUNX1 *over-expression in the TEL/AML1-positive group by microarray experiments and q-PCR. Expression and cytogenetic data from patients 9 and 17 indicate that RUNX1 over-expression is not due to the expression of the *TEL/AML1 *(*ETV6/RUNX1*) fusion gene, driven by the *TEL *promoter, but to the native *RUNX1 *gene. An increased *RUNX1 *copy number was also found in one third of the *TEL/AML1 *patients, and this may explain, at least in part, *RUNX1 *over-expression. Gene amplification is a common mechanism of oncogene deregulation, which occurs with *RUNX1 *through chromosome 21 polysomy, by the presence of a *RUNX1 *tandem repeat on der(21) or with additional *RUNX1 *copies on extra-chromosomal elements [24]. The over-expression of the *RUNX1 *gene with no apparent amplification of the *RUNX1 *locus also suggests that there may be cryptic amplification undetectable by FISH-analysis or deregulation of the *RUNX1 *promoter. Conversely, promoter silencing or gene deletion despite over-representation of chromosome-21 could explain low expression of the RUNX1 gene. *RUNX1 *over-expression appeared to be characteristic of the *TEL/AML1*-positive patient group. Indeed, all patients with *RUNX1 *over-expression clustered together, including the patient 9, who had four copies of *RUNX1 *but no *TEL/AML1 *fusion. By contrast, *TEL/AML1-*positive patient 17, who presents no RUNX1 over-expression, did not segregate with the *TEL/AML1*-positive group. It is possible that the expression levels of *RUNX1 *could explain the clinical heterogeneity of t(12;21) ALL cases. Indeed, it has been suspected that a *RUNX1 *gene copy number of four is associated with ALL with good prognosis. By contrast, whereas the amplification of *RUNX1 *to a copy number greater than four, which has been estimated to be the case in 2% of all pediatric ALL and particularly those with no TEL/AML1 chromosomal aberration, may be characteristic of a subtype of B-ALL associated with a poor prognosis [25,26]. If these findings were confirmed, the *TEL/AML1 *fusion transcript and *RUNX1 *expression level data could be used as stratifying therapeutical markers, with possible prognostic value.

## Conclusion

Gene expression analysis of *TEL/AML1 *ALL identified five enriched gene ontology (GO) categories: cell differentiation, cell proliferation, apoptosis, cell motility and response to wounding, associated with fourteen genes able to cluster the *TEL/AML1 *sub-group (*RUNX1, TCFL5, TNFRSF7, CBFA2T3*, *CD9*, *SCARB1, TP53INP1, ACVR1C, PIK3C3, EGFL7*, *SEMA6A, CTGF, LSP1, TFPI*). These results, based on a small cohort, but validated by two independent data sets, should serve as a basis for a better understanding of TEL/AML1 pathogenesis and the biology of late relapse.

## Methods

### Patients

Bone marrow leukemia cells were obtained at diagnosis, with informed consent, and after agreement of the Ethics Committee of Rennes Hospital (Rennes, France). For each patient, the bone marrow blast-cell level was > 80%. CD19, CD10 and CD79A were expressed in all samples and none were classified as biphenotypic ALL. Each sample was analysed by conventional karyotyping and tested for the presence of *E2A/PBX1, TEL/AML1, BCR/ABL *and *MLL *rearrangement by RT-PCR. Positive results were confirmed by *in situ *fluorescence analysis. Independent of the presence of a *TEL/AML1 *rearrangment, children were assigned to risk and treatment groups (standard risk or high risk) according to the FRALLE 2000 protocol (France Acute Lymphoblastic Leukemia de l'Enfant), with an initial risk-adapted stratification of treatment at diagnosis based on age (1 year ≤ age < 10 years, or ≥ 10 years), white blood cell count ( < 50,000/mm^3^, or ≥ 50,000/mm^3^), involvement of the central nervous system (CNS) and cytogenetic data. The early *in vivo *response to treatment was further assessed as the response to steroid and chemotherapy treatment, and by molecular measurement of the minimal residual disease, using polymerase chain reaction amplification on day 35 of treatment to detect the presence of clone-specific immunoglobulin and T cell receptor-gene rearrangements.

### RNA isolation, and reference RNA

Mononuclear cells were isolated from bone marrow (2 ml samples, i.e. 5 million cells) by successive centrifugations through MLS medium (Eurobio, Courtabeuf, France). Isolated leukocytes were immediately stored at -80°C in 1 ml of RNA-PLUS solution (Qbiogene, Strasbourg, France). Total RNA was recovered using a Qiagen RNeasy column (Qiagen, Hilden, Germany) according to the manufacturer's instructions. In-column DNase treatment was carried out before eluting the RNA to ensure the absence of genomic DNA. Recovered RNA was quantified using a Nanodrop 1000 spectrophotometer (Nanodrop Technology^®^, Cambridge, UK) and RNA integrity was assessed using a 2100 Bioanalyser (Agilent, Palo Alto, CA, USA). RNA samples with an RNA integrity number (RIN) greater than 9 were used for further analysis. We used an equimolar-pooled mixture of each test RNA sample for the reference RNA for two-color microarray technology.

### Targets preparation and microarrays hybridization

RNA samples (test and reference) were labeled using the Agilent low RNA input fluorescent linear amplification Kit (p/n 5184-3523) according to the manufacturer's instructions. To avoid confounding by extraneous factors, all the experiments were performed with a single batch and processed by one technician on the same day for each step. Briefly, 500 ng of total RNA was reverse transcribed. Amplification and labeling were performed by T7-polymerase *in vitro *transcription, to give fluorescent-labeled cRNA. Test and reference cRNAs were labeled with Cyanine-5 and Cyanine-3 CTP dyes, respectively (10 mM, PerkinElmer, Norwalk, CT). The dye incorporation rate was assessed with a Nanodrop^® ^ND-1000 spectrophotometer and was found to be between 1.2 and 1.4 pmol/μl. Hybridization was carried out using the Agilent oligonucleotide microarray *in situ *hybridization plus kit (p/n 5184-3568), following the manufacturer's instructions. Briefly, 750 ng of test sample cRNA was mixed with 750 ng of reference sample cRNA in the presence of target controls. This solution was subjected to fragmentation (30 min at 60°C) and then hybridization on 44K Human Whole-Genome 60-mer oligo-chips (G4112A, Agilent Technologies) in a rotary oven (4000 rpm, 60°C, 17 h). Slides were disassembled and washed in solutions I and II according to the manufacturer's instructions, and dried using a nitrogen-filled air gun before scanning.

### Data acquisition and processing

Microarrays were scanned with a dynamic autofocus microarray scanner (Agilent dual laser DNA microarray scanner -G2566AA, Agilent technologies, Palo Alto, CA, USA), using Agilent-provided parameters (Red and Green PMT were each set at 100%, and scan resolution was set to 10 μm). The Feature Extraction Software v7.5 (Agilent technologies, Palo Alto, CA, USA) was used to extract and analyse the signals. Agilent-provided settings were used except for subtraction of the local background and adjustment of the global background. Poor quality features that were either saturated in the two channels (50% of pixels > saturation threshold) or non-uniform were flagged. Only those features with a signal-to-noise ratio (SNR) of up to 2.6 in at least one channel and significantly different from the local background (two sided Student's t-test < 0.01) were used for further analysis. The mean signal ratio of the two fluorescent intensities (Cy-5 cRNA test /Cy-3 pooled cRNA) is expressed as a logarithm (base 2), providing a relative quantitative gene expression measurement between two samples. For subsequent analysis, we used mean-centered log_2 _of the normalized (linear & lowess method) sample:reference ratio.

The accuracy of microarray results was assessed by comparing the global gene expression levels of each chip using box-plot analysis. Each box-plot was centered on zero with comparable dynamic intensities, revealing the technical homogeneity of the overall experiment (data not shown). Furthermore, technical duplicates with 25% of the Set-A cRNA samples were used to assess the reproducibility of the array.

See additional  files in microarray database "Gene Expression Omnibus" : accession number GSE 9170.

### Selection and profiling of differentially expressed genes

Spot signals with a PvalueLogRatio < 0.01 were selected from each array and used for further analysis. Missing values, due to flagged signals, were replaced using the K-nearest neighbors calculation method with k = 10, but only when there were less than 30% of values missing per gene.

We used two-class unpaired significance analysis of microarrays (SAM) to select genes that were differentially expressed in *TEL/AML1*-positive patients and *TEL/AML1*-negative patients [27]. Genes selected following 1000 permutations were those with expression that was more than 1.7-fold different from the mean expression. We also used a Qvalue < 0.02 as an additional discriminating parameter. Benjamini and Hochberg false discovery estimation was applied to validate our data because of the small size of the cohort. We used the agglomerative hierarchical clustering approach (average or complete linkage clustering using Euclidean distance as distance metric) in the TIGR Mev 3.1 software [28] to represent SAM-selected genes [29] and assessed reproducibility by using a resampling-based procedure (bootstrapping for 100 iterations).

### Functional annotation

The genes identified by the SAM selection and hierarchical clustering representation were functionally annotated using the WebGelstalt toolkit (WEB-based GEne SeT AnaLysis Toolkit, University of Tennessee and Oak Ridge National Laboratory)[30]. WebGelstalt includes information from the Gene Ontology Tree Machine software [31] and queries were made with lists of official gene symbols (approved by the HUGO Nomenclature Committee). We retrieved enriched GO terms (i.e. GO terms with a significantly higher than expected number of associated genes) from the GOTree module (displayed as a Directed Acyclic Graph) and KEGG biochemical pathways (displayed as a KEGG table) using a hyper-geometric statistical test. Selected enriched GO terms were those that comprised at least two genes with a Pvalue < 0.05 by comparison to the pre-stored Agilent G4112A gene set. The annotation concerned only the biological process.

### Quantitative reverse transcription (RT)-PCR

Real-time PCR was carried out in sealed 96-well micro-titer plates using the SYBR™ Green PCR Master Mix (Applied Biosystems^®^), according to Applied Biosystems gene amplification specifications (40 cycles of 15 sec at 95°C and 1 min at 60°C). We analysed gene expression using the ABI Prism 7000 sequence detection system (Applied Biosystems^®^) and evaluated the results using the associated software (version 1.2.3, Applied Biosystems^®^). *ABL1 *RNA was chosen as an internal positive control, because it showed no significant variation in our experiments. The relative amounts of the gene transcripts were determined using the Ct method, as described by the manufacturer. The mRNA levels are expressed with respect to the mean Ct values of all samples. Each PCR experiment was carried out in triplicate. Data are given as mean expression (with the SD) for each gene per group (*TEL/AML1 *and non *TEL/AML1*).

The following forward (F) and reverse (R) primers were designed using the Primer Express™ software (version 2.0-PE Applied Biosystems^®^):

F-ABL1: 5'-CGCTCATCACCTAAACTTGTACTTT-3';

R-ABL1: 5'-CTGTAAGAACCGCATAAAACGA-3';

F-CBFA2T3: 5'-TGAACTCGACATTGACGATCG-3';

R-CBFA2T3: 5'-TCAGGAAGGGAATGACAAACG-3';

F-CD9: 5'-CAACAAGCTGAAAACCAAGGA-3'

R-CD9: 5'-CAAACCACAGCAGTTCAACG-3'

F-EGFL7: 5'-TTGCCAGTCAGATGTGGATGA-3'

R-EGFL7: 5'-ACTCTGTGTGCCCAAGGGAG-3'

F-LSP1: 5'-AGGGGGAGCAAGAGGACA-3'

R-LSP1: 5'-CCCCTCCTTGCTCAGACTC-3'

F-PIK3C3: 5'-GCCTTGGAACTTCTGGGAAAA-3';

R-PIK3C3: 5'-CAACAGCATAACGCCTCACAG-3';

F-RUNX1: 5'-ACAAACCCACCGCAAGTC-3'

R-RUNX1: 5'-catctagtttctgccgatgtctt-3'

F-TCFL5: 5'-GCGCAGAATCCGCATTTG-3';

R-TCFL5: 5'-TCAGGAATGCTGTGGTCCACT-3'

F-TNFRSF7: 5'-AGGGACAAGGAGTGCACCG-3';

R-TNFRSF7: 5'-AAGGTAAGTGGGTGGGCTGAG-3';

F-TP53INP1: 5'-GCATGTCTGTCTATGCTGTGC-3'

R-TP53INP1: 5'-TTCATTTTGAGCTTCCACTCTG-3'

Primer specificity was assessed from their mono-phase dissociation curves and all pairs presented comparable efficiencies (data not shown).

### Karyotype and FISH analysis

At the time of the diagnosis, chromosome analyses were performed on bone marrow samples using a RHG-banding technique. Karyotypes were designated according to the International System for human Cytogenetic Nomenclature (ISCN, 1995).

Fluorescence *in situ *hybridization (FISH) analyses were performed when a t(12;21) was suspected (either RT-PCR or karyotype), according to the manufacturer's protocol using the Vysis LSI *TEL/AML1 *bicolor probe (Abbott, Rungis, France).

## Authors' contributions

VG interpreted and analyzed the data, drafted of the article, made critical revisions, a substantial intellectual contribution, provided study materials/patients, and collected and assembled the data. AGR provided major contribution concerning biological and process information and analyzed the data. MDT contributed to the statistical analysis of the data, making a major intellectual contribution. VS contributed to data acquisition and processing, interpreted and analyzed the data. SM and AM interpreted and analyzed the data. BLS and CH provided study materials/patients, and collected and assembled the data. CS and ELG provided study materials/patients and critical revisions. ALT provided platform support and critical revisions. CB interpreted and analyzed the data, and provided study materials/patients. JYLG and JM contributed to the concept and design of the study, provided critical revisions and made major intellectual contributions. MDG contributed to the concept and design of the study, interpreted and analyzed the data, contributed critical revisions and intellectual input, gave final approval, and collected and assembled the data.

## Competing interests

The author(s) declares that there are no competing interests. 
